# Pediatric Hemangiomas in the Female Genital Tract: A Literature Review

**DOI:** 10.3390/diseases12030048

**Published:** 2024-02-28

**Authors:** Lucia Merlino, Agnese Immacolata Volpicelli, Franco Anglana, Giulia D’Ovidio, Mattia Dominoni, Marianna Francesca Pasquali, Barbara Gardella, Paolo Inghirami, Pietro Lippa, Roberto Senatori

**Affiliations:** 1 Department of Medical-Surgical Sciences and Biotechnologies, Sapienza University of Rome, 00161 Rome, Italy; lucia.merlino@uniroma1.it; 2 Department of Maternal Infantile and Urological Sciences, Sapienza University of Rome, 00161 Rome, Italy; volpicelli.1800695@studenti.uniroma1.it (A.I.V.); g.dovidio@uniroma1.it (G.D.); 3 Vulvar Pathology Study Center, 00161 Roma, Italy; franco.anglana@gmail.com (F.A.); paolo.inghirami@gmail.com (P.I.); mattia.dominoni01@universitadipavia.it (P.L.); robertosenatori@gmail.com (R.S.); 4 Department of Clinical, Surgical, Diagnostic and Pediatric Sciences, University of Pavia, 27100 Pavia, Italy; matti.domino@gmail.com (M.D.); barbara.gardella@gmail.com (B.G.); 5 Department of Obstetrics and Gynecology, IRCCS Foundation Policlinico San Matteo, 27100 Pavia, Italy

**Keywords:** pediatric hemangioma, vaginal hemangioma, vulvar hemangioma, hemangioma of lower genital tract

## Abstract

Background: Hemangiomas are aberrant proliferations of blood vessels and the most frequent benign pediatric soft tissue tumors. Although they are common, genital localization is rare. This study aimed to assist doctors in the diagnosis, management, and treatment of pediatric vulvovaginal hemangiomas by conducting a review of the literature. Methods: We conducted a literature review including papers published between August 2009 and May 2023. Results: While most hemangiomas are usually indolent and resolve with time, in some cases, especially cervicovaginal and uterine ones, they might present with severe symptoms like heavy bleeding and need further instrumental investigation for diagnosis, like CT or MRI. As for the treatment, many options are available, with medical therapy or expectant management being the first choice. Conclusions: Vulvovaginal pediatric hemangiomas are rare and require more research on how to detect and manage these lesions, especially the symptomatic and the psychologically impacting ones. For the time being, treatment should be personalized based on the patient’s situation and clinician’s expertise.

## 1. Introduction

Hemangiomas are benign vascular tumors that can arise on the skin or in internal organs. They are caused by stem cells that, in response to various stimuli, can proliferate and differentiate into a variety of cell lines, including mesenchymal stem cells, endothelial progenitor cells, neuroglial stem cells, and hematopoietic stem cells [1,2].

The International Society for the Study of Vascular Anomalies (ISSVA) has proposed various classifications of these vascular conditions, stressing the difference between “real” hemangiomas and vascular malformations; in fact, while the former are true neoplasms of endothelial cells, the latter are localized structural anomalies of vascular morphogenesis brought on by malfunction in embryogenesis and vasculogenesis that increase as a result of endothelial cell hyperplasia [3]. 

Hemangiomas are the most frequent benign pediatric soft tissue tumors, affecting 12% of this population. The head and neck region accounts for 60% of cases, with the trunk following at 25%, and the extremities at 15%; the genital area is rarely affected. They are classified as congenital hemangiomas (CHs) and infantile hemangiomas (IHs). CHs represent 30% of all hemangiomas; at birth they are clinically evident as completely grown lesions that either involute quickly in the first year of life or never display involution, and they have equal male and female prevalence. Conversely, IHs are more common (70%), occur at 2–8 weeks of age, grow rapidly for about 6–12 months, and then have a slow involution, with 90% of them disappearing by the age of 9 years. 

For IHs, the main risk factors are represented by low birth weight, multiple pregnancies, female sex, white population, higher maternal age, progesterone therapy, and positive family history [4]. They typically occur in childhood and usually undergo involution, whereas vascular malformations are typical in adulthood and are divided into arterial, venous, capillary, lymphatic, or mixed. The most prevalent of them are venous malformations (VMs), once known as “cavernous hemangiomas” [3,5].

Infantile hemangiomas are classified by depth:

- Superficial: located at the level of the superficial dermis, usually red;
- Deep: located in the deep dermis and subcutaneous tissue, usually blue;
- Combined: both superficial and deep components present.

And by anatomical aspects:

- Localized: well-defined lesion that seems to originate from a central point;
- Segmental: involving a plaque-like anatomical region, usually measuring > 5 cm;
- Indeterminate: neither clearly localized nor segmental;
- Multifocal: present at disparate sites.

Hemangiomas are also classified into high and low risk, taking into account the clinical characteristics of the lesions:

- Life-threatening: located along the respiratory tract, which can cause obstruction and death due to respiratory complications;
- Functional impairment: located along the oral fissure or affecting the eyes, which can be the cause of a functional limitation;
- At risk of ulceration;
- Associated with structural anomalies (ex. lumbar syndrome);
- Hemangiomas which, due to their location, represent a disfigurement for the patient [6].

Hemangiomas are usually clinically silent lesions that are occasionally found during clinical examination, even if sometimes they can give rise to profuse bleeding that requires medical intervention [1].

**Figure 1:**
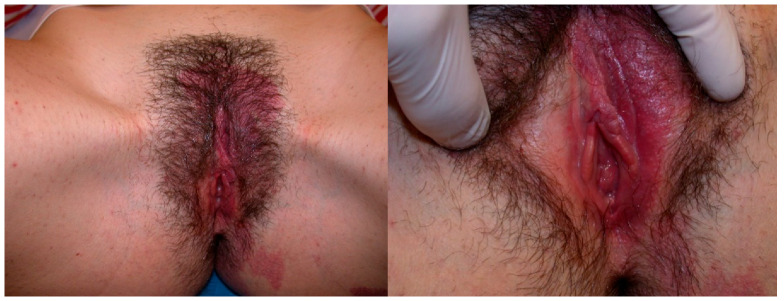

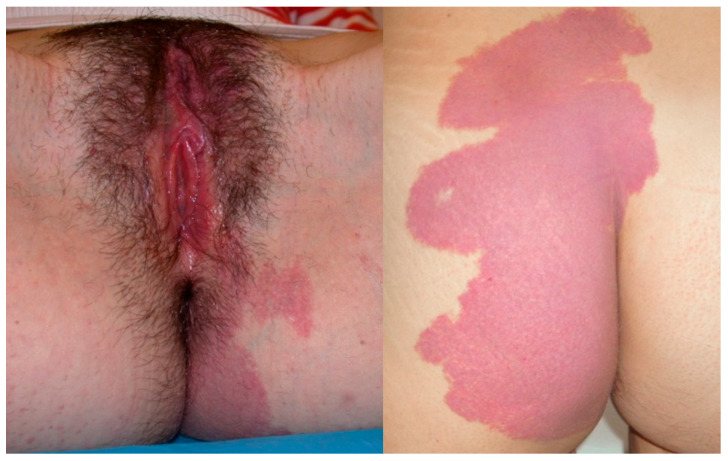
An 18-year-old girl presented with a large vulvar hemangioma involving the labia minora that has continued into the vestibule, extended to the perianal area and to the left buttock and back.

**Figure 2:**
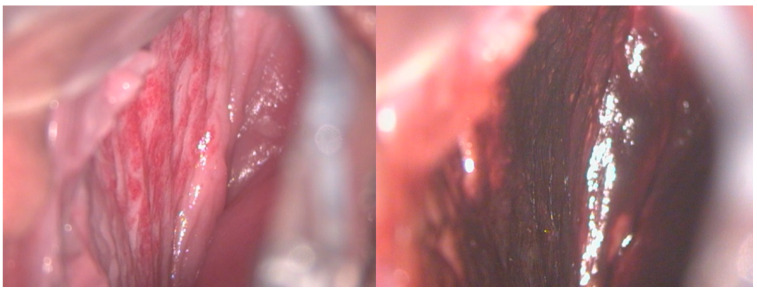
A 17-year-old girl reported significant vaginal blood loss, not related to sexual intercourse. A colposcopic examination highlighted the presence of hemangioma of the right vaginal wall, resulting in a negative Lugol test, used as a tool for differential diagnosis.

**Figure 3:**
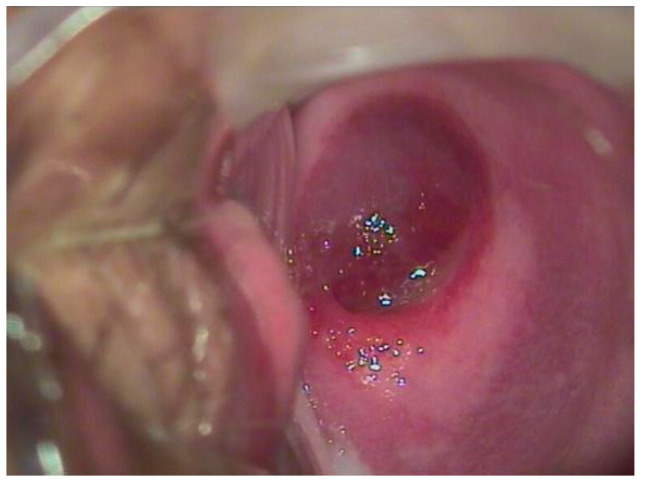
A 17-year-old patient complained of significant blood loss and intimate discomfort. The gynecological examination with a speculum revealed a hemangioma of the cervix.

Concerning the female genital tract, shown below are cases of hemangiomas of the uterus, vagina, and cervix and, regarding the urogenital part, of the urethra and the bladder (Figure 1, Figure 2 and Figure 3). 

Due to their rarity, there is no treatment that is widely accepted; nowadays, the main options are represented by propranolol, topic corticosteroids, and surgical excision, followed by laser therapy, embolization, radiotherapy, intermittent pneumatic compression, and continuous compression in the treatment of symptomatic hemangiomas. Also, expectant management could be considered in asymptomatic lesions. Therefore, the symptoms produced, the localization of the lesion, and the institution’s level of experience handling such cases all influence the management of this condition [7].

The aim of this study was to conduct a review of the literature to analyze the clinical characteristics of pediatrics vulvovaginal hemangiomas, in order to help clinicians in the diagnosis, management and treatment of these lesions. 

## 2. Materials and Methods

The most important medical databases, such as PubMed, Cochrane Database of Narrative Reviews, EMBASE, and Web of Science, were consulted in order to obtain a review of the literature regarding pediatric vulvovaginal hemangiomas. The following keywords were used in combination as a search strategy: “vaginal hemangiomas”, “vulvar hemangiomas”, “lower genital tract hemangiomas”, “infant”, and “childhood” including pluralization and US English/United Kingdom English spelling variations and suffixes/prefixes.

All online publications from August 2009 to May 2023, including case series, literature reviews, and prospective and retrospective trials, were considered for our study. Using the Preferred Reporting Items for Systematic Reviews and Meta-Analyses (PRISMA) literature selection method, we conducted a systematic search. Two authors (L.M. and R.S.) separately searched the manuscripts’ reference lists to incorporate the literature into the review. Articles documenting the characteristics and management of vulvar hemangiomas were given special consideration. 

Studies were considered eligible and included in the analysis if they fulfilled the following criteria: (I) histological features of vulvar hemangiomas; (II) characteristics of pediatric hemangiomas; and (III) management of lower genital tract hemangioma in pediatric age.

After the chosen articles were evaluated as full-text literature, the data were tallied.

The exclusion criteria were the following: (I) case reports that were deemed less noteworthy on this subject; (II) medical abstracts and publications produced in languages other than English; (III) multimedia resources pertaining to the study’s goal: conferences, editorials, and preliminary research in animal models.

In accordance with the Cochrane Handbook for Systematic Reviews of Interventions [8], two investigators (L.M. and R.S.) independently, assessed the risk of bias for each selected study to ensure validity and overcome eventual selection, performance, detection, attrition, and reporting bias. 

Bias in the research as well as risks associated with the funding source and the authors’ conflicts of interest were evaluated. Discussions were employed to settle actual disputes.

The researchers (L.M. and R.S.) separately extracted the data and evaluated the form for data extraction.

Because the included study designs and outcome measures were heterogeneous, a meta-analysis could not be performed.

**Figure 4:**
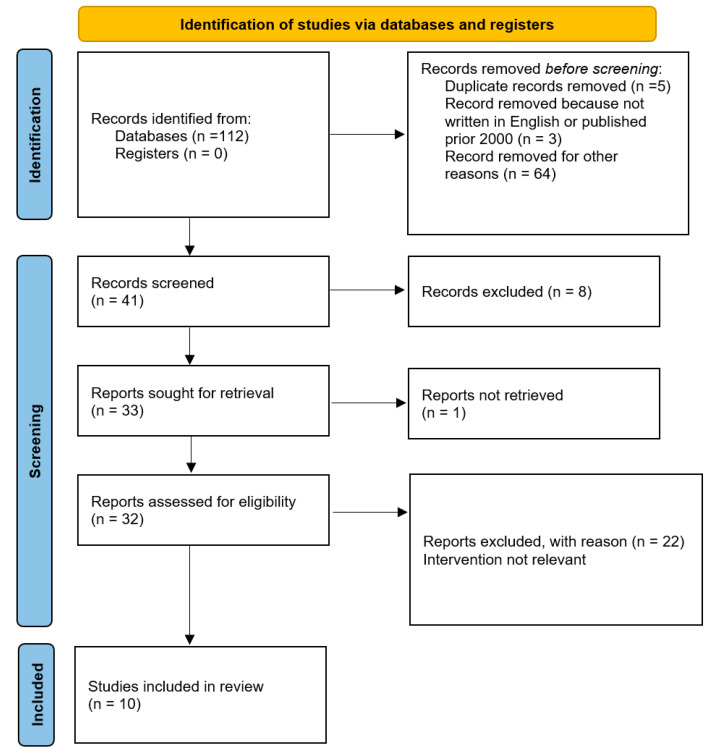
PRISMA flow diagram of study selection process.

A total of 112 studies were found for our review using the search technique. Duplicate papers that were listed in multiple databases and unrelated publications were disregarded in our study. Additionally, we eliminated all articles that were not published in English or that were published prior to 2000. The papers were then screened by title and/or abstract. In the end, 10 studies that examined potential biomechanical and microscopic alterations that brought on pediatric lower genital tract hemangiomas were compared and included (Figure 4).

## 3. Results

The analysis of the data highlighted that the present literature on vulvovaginal hemangiomas in childhood and adolescence is limited to a small number of case reports, reviews, or clinical retrospective or prospective papers.

The main characteristics of the 10 papers retrieved and examined are summarized in Table 1. They are the result of investigations conducted with the indications detailed in Materials and Methods. 

In the papers analyzed, we found seven case reports of patients who ranged in age from 5 months to 13 years old, with an early onset of symptoms, when present. Among the cases, one was asymptomatic and the hemangioma was incidentally diagnosed, two reported swelling in the genital area, and four presented with vaginal bleeding. 

While mild or no symptoms were associated with vulvar localization, moderate to severe symptoms were associated with one case of vaginal hemangioma, one case of uterine hemangioma, and two cases of cervical hemangiomas, one of which also developed an intrauterine localization. The last case, described by Gada et al., presented with irregular bleeding at an early age, with the diagnosis of IH of the cervix that was surgically treated. After that, with the advent of menarche, the patient continued experiencing heavy menstrual bleeding, poorly controlled with oral contraception and IUD, and was evaluated for infertility. The US showed only one area suggestive of adenomyosis, and the hysteroscopy performed was negative. The patient sought definitive therapy at the age of 28 due to recurrent vaginal bleeding. Following counseling on the advantages and disadvantages of uterine artery embolization, the patient underwent a straightforward vaginal hysterectomy with ovarian preservation. No hemangiomas were observed in the vagina or encountered during uterine excision. Pathology of the uterus showed a diffusely involved hemangioma in the posterior myometrium. The patient did not require a blood transfusion, unlike the 6-year-old girl from Ganti et al., who experienced significant blood loss due to IH of the cervix. In only one case, concomitant abdominal pain and constipation were present, but targeted diagnostic investigations were performed and found to be negative. 

Concerning the treatment options, both surgical and medical therapy were used, while no cases of laser therapy were retrieved. Other than the case of Gada et al. previously described, three patients, one with a highly vascularized focal intrauterine intracavitary lesion and two with superficial vulvar hemangiomas, benefited from surgical excision with a complete and immediate recovery. In one patient with mild to moderate vaginal bleeding, Jackson et al. decided on expectant management, with a decrease in symptoms and size of the lesion after 2 months; conversely, Ganti et al. managed to control the heavy vaginal bleeding of a vaginal IH with oral propranolol and blood transfusion, leading to a significant reduction in the size of the lesion and the complete resolution of symptoms. The asymptomatic 11-year-old girl with vulvar hemangioma was followed over time and has had no other problems. 

## 4. Discussion

Hemangiomas are aberrant proliferations of blood vessels, which may occur in any vascularized tissue of the body. They are frequently discovered in the subcutaneous and dermal tissues, while they are very uncommon in the female genital tract. When present, these benign tumors are the most prevalent in infants and often resolve by adolescence [7].

In the literature, there are only four documented cases of IHs affecting the female internal genital tract: a single case in the vagina, two in the uterine cervix, and one in the corpus uteri. Regarding vulvar hemangiomas, a bit more literature is available, sometimes associated with multifocal or segmental IHs in the previously described syndromes.

The pathogenesis of IHs is not clear, but an important role is certainly played by pluripotent stem cells which respond incorrectly to stimuli supplied by hypoxia and by the renin angiotensin aldosterone system [4]. A placental origin has also been hypothesized because the expression of placental markers has been observed in the endothelium of infantile hemangiomas [18].

In this regard, many studies have found in IHs a GLUT-1 (Glucose transporter-1) positivity. GLUT-1 is a sensitive and specific marker for IHs that cannot be identified in other vascular anomalies that fall under the histopathological differential diagnosis, for example, varices, arteriovenous malformations, and lymphatic malformations. Additionally, proliferating and involuting IHs are characterized by other unspecific markers, such as VEGF, bFGF, collagenase IV, and urokinase, which are not expressed by other vascular malformations [3,13]. 

In non-cutaneous cases, when inspection and vaginoscopy are not conclusive, imaging can complement the diagnosis, based on different clinical pictures. For example, Ralph et al. performed a CT scan and an MRI to assess the cause of vaginal bleeding, thus discovering a highly vascularized intrauterine intracavitary lesion. 

These lesions are characterized by peculiar signs which can help to confirm the diagnosis in challenging cases and with presurgical planning. For instance, they are highly vascularized, but with no arteriovenous shunting on the US; can appear as lobular soft tissue mass, intensely staining on a CT scan; and MRI shows an isointense (T1), hyperintense (T2), homogeneously enhancing soft tissue mass, with flow voids within and surrounding IHs [10].

While most of these benign tumors are usually indolent, in some cases they might present with severe bleeding or result in emotional or functional impairment. Table 2 shows the list of possible causes of bleeding in pre-adolescent girls, which enter into differential diagnosis with hemangiomas. For this reason, it is important to know that various treatment options are available and, since spontaneous regression of the tumor is possible, expectant management should be considered as an option [7]. 

Nowadays, the basis of systemic therapy is propranolol, a non-selective beta blocker, on a level of evidence of 1A. Before starting treatment, it is advisable to carefully analyze the patient’s clinical history and vital signs. In children born preterm, with low birth weight or a history of hypoglycemia, it is recommended to perform a basal blood glucose measurement [19,20]. Since the medication is contraindicated in situations of bundle branch blockages and arrhythmias, an electrocardiogram should be performed in individuals with a positive anamnestic history of cardiac or connective tissue disorders. The molecule functions on multiple fronts, including vasoconstriction, prevention of angiogenesis, induction of apoptosis, inhibition of nitric oxide synthesis, and modulation of the renin-angiotensin system, though the exact reason for its usefulness is unclear [21].

More specifically, firstly propranolol induces a fast vasoconstriction, which is associated with the usual color shift from pink to violaceous that occurs in the first one to two days of medication. Secondly, IH growth arrest appears to be associated with the downregulation of proangiogenic growth factors such as matrix metalloproteinases 2 and 9, vascular endothelial growth factor, and basic fibroblast growth factor. Thirdly, it has been suggested that acceleration of endothelial cell apoptosis induction, which is known to happen during natural involution, causes IH regression [10]. 

The recommended dose is 2 and 3 mg/kg per day in patients at risk; however, it is recommended to start with 1 mg/kg per day and evaluate the body’s response before raising the dose to the target [21]. 

Other useful beta blockers are atenolol and nadolol; these have the advantage of fewer side effects and longer half-life compared to propranolol. Atenolol is a selective β1-antagonist with a lower risk of bronchospasm and hypoglycemia. The recommended dose is 1 mg/kg per day for IHs [20]. For smaller, more cosmetically sensitive lesions, topical timolol 0.5% gel-forming solution formulation is an alternative. In certain situations, it can be used as an adjuvant to systemic therapy, either to delay the start of systemic therapy or to expedite the tapering of propranolol with a subsequent switch to timolol to prevent posttreatment rebound growth [10].

The difficulty with these medications is that they increase the release of nitric oxide and nitrogen peroxide at the hypothalamus level; thus, they can have detrimental neurological consequences; in fact, further pharmacokinetic studies are needed [22]. 

Corticosteroids have long been the only therapy used for hemangiomas; their use was gradually reduced when beta blockers were introduced. At present, however, in the event that there are contraindications to the use of propanol or in non-responders, prednisone and prednisolone should be administered. The recommended initial dose is 2–3 mg/kg/day, and the duration of treatment varies from 9 to 12 long cycles [23]. In the case of high-risk hemangiomas, the combined therapy of propanol and corticosteroids can be chosen [24]. In our literature search, two cases were treated with propranolol, causing stabilization of or a reduction in the lesion, and one case with prednisolone, resulting in two recurrences which, in the end, required surgical treatment.

For small proliferating IHs, intralesional steroid injections may be beneficial. They may also stabilize growth and reduce the size of IHs, obviating the need for surgical intervention or systemic therapy.

Corticosteroids can have some adverse effects, which include cushingoid facial features, insomnia, irritability, gastroesophageal reflux, and temporary growth retardation, as well as more severe but uncommon side effects including osteoporosis, obstructive hypertrophic cardiomyopathy, adrenal insufficiency, and hypertension [17]. 

Besides this first-line therapy, there are other molecules, like captopril and sirolimus, whose mechanism of action in this area has yet to be further investigated [21]. 

In the past, lasers were somewhat helpful for IH primary care. However, the introduction of propranolol as a major treatment for IH has narrowed the scope of laser control to very specific uses. For instance, a common use is the intralesional application of the Nd:YAG laser for medically refractory lesions, or of the PDL laser for persistent cutaneous lesions that do not completely resolve with pharmaceutical care, either alone or in combination with beta blockers as combined primary management. Many studies have demonstrated that the combination of laser therapy and a beta blocker (ex. propranolol) systemically has proven to be superior to monotherapy [25,26].

In our review, no cases of laser therapy were retrieved. This could be explained by the fact that, except for one study, all the others are recent and, as the literature suggests, since the advent of propranolol and other medical therapies, laser treatment has taken a back seat. In addition, it should be kept in mind that it is a more expensive procedure and not found in all centers. 

Eventually, for superficial hemangiomas, topical treatment is also recommended, specifically with the use of timolol maleate 0.5% applied twice daily, as mentioned above, which has replaced the use of corticosteroids [27]. Timolol is a non-selective beta blocker that has been shown to be even more effective in the treatment of superficial hemangiomas than propranolol and with fewer adverse effects [28]. 

Even if medical therapy remains the first choice, sometimes procedural interventions are needed. 

To treat focal, bulky IHs, intralesional injection of corticosteroids (triamcinolone and/or betamethasone) or bleomycin may be utilized in addition to oral β-blockers, or in non-responders. 

Cryotherapy has proven to be effective in both superficial and deep IHs. 

Surgery is usually recommended for life- or function-threatening IHs, for example, hemangiomas of the airways or causing massive bleeding, or in cases of unsuccessful or contraindicated medical therapy. This is because hemangiomas usually have an expansive rather than an invasive growth pattern, thus displacing the neighboring structures; this requires the surgeon to radically remove them. Sometimes, when there are limit situations with uncontrolled bleeding or with expansile masses causing organ compromise or cardiac failure, embolization may be an option. In the cases analyzed, surgical excision was used both as a second-line treatment after the failure of medical therapy, and as a first-line treatment in a child with intrauterine hemangioma causing significant vaginal bleeding and in a 13-year-old child with a circumscribed vulvar lesion [21].

**Figure 5:**
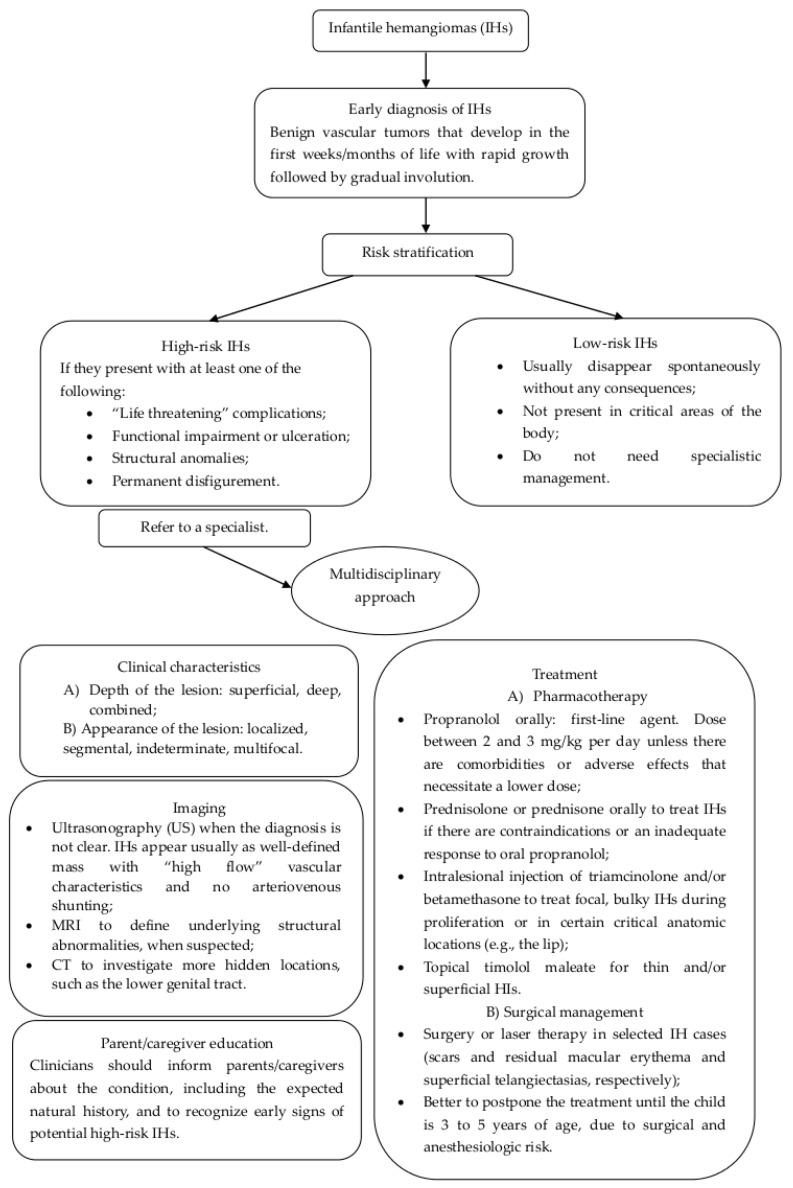
Flow chart for the management and treatment of infantile hemangiomas of lower genital tract.

In Figure 5, we present a flow chart that may be useful to clinicians to identify the best diagnostic procedures and treatments in order to adequately manage pediatric hemangiomas.

## 5. Conclusions

Hemangiomas are the most common benign tumors of childhood, and the most frequent subtype are infantile hemangiomas (IHs), accounting for 70% of the total. Although they are common, genital localization is rare. When the possibility of hemangioma arises, it is essential to exclude other pathologies which may enter into the differential diagnosis, since prognoses and therapeutic methods are different.

Hemangiomas are mostly asymptomatic and resolve with age, but sometimes they can result in local tissue damage, ulceration, infection, bleeding, functional impact, and pain. Because of the rarity of the condition, there is no universally accepted method of treatment; therefore, its management should be personalized based on the clinician’s experience and on the options available. At the same time, additional research on hemangiomas of the female lower genital tract is needed.

## Figures and Tables

**Table 1:** Cases in the scientific literature that describe the diagnosis, management, and follow-up of pediatric patients with hemangiomas of the lower genital tract.

Variables	Main Findings
General characteristics of presentation of vulvovaginal pediatric hemangiomas [9,10,11]	- IHs are the most frequent soft tissue tumors in children as well as the most common benign vascular tumors, which are distinguished by a distinct tripartite growth cycle of proliferation, plateau, and involution. - Although IHs are common, they are rare in the genital area, which accounts for about 1% of cases. - Infants that develop this pathology are more likely to be female, preterm, of white non-Hispanic ethnicity, and born to older mothers.
Clinical presentation and symptoms [11,12,13,14,15]	- Hemangiomas of the lower genital tract can be asymptomatic or present with a variety of symptoms that often can lead to a misdiagnosis. Most of them will involute without intervention. - Vulvar hemangiomas can appear either as solid, pedunculated, exophytic tumors or as blue-purple, compressible, non-expansile, and non-pulsatile masses on the labia. - Urogenital hemangiomas (urethra, cervix, clitoris) often come with a negative external physical examination and US and/or MRI are needed to confirm the diagnosis. In the presence of hematuria or vaginal bleeding in a pediatric patient, urethral or cervix hemangioma should be considered in the differential diagnosis. - Cervicovaginal and uterine hemangiomas can lead to heavy or irregular bleeding that could be mistaken for hormonal or structural anomalies. If not detected and treated in time, uterine IHs could lead to infertility.
Comorbidities associated with pediatric hemangiomas [9,10,14]	- Multifocal and segmental IHs have been shown to be more associated with congenital malformations compared to the general population, even though they occur in a very small proportion of cases. The most frequent syndromes described are - PELVIS (perineal hemangioma, external genitalia malformations, lipomyelomeningocele, vesicorenal abnormalities, imperforate anus, and skin tag); - SACRAL (spinal dysraphism, anogenital anomalies, cutaneous anomalies, renal and urological anomalies, associated with lumbosacral hemangioma); - PHACES (posterior fossa malformations, hemangiomas, arterial anomalies, coarctation of the aorta and cardiac defects, eye abnormalities, and sternal or ventral defects); - LUMBAR (lower body congenital infantile hemangiomas and other skin defects; urogenital anomalies and ulceration; myelopathy; bony deformities; anorectal malformations and arterial anomalies; and rectal anomalies).
Immunohistochemical characteristics [13,16]	- Immunohistochemical examination can help confirm the diagnosis; in fact, IHs have a diffuse positive staining for glucose. In particular, there are two markers: transporter protein-1 (GLUT-1), which is expressed by endothelial cells within IHs and differentiates IHs from other vascular lesions, and Wilms’ tumor (Wt-1), which stains for IHs but not AVMs (arteriovenous malformations).
New perspectives on diagnosis [10,11,14]	- Except for vulvar localization, the other IHs of the lower genital tract are often not diagnosed with an initial visual inspection of the anogenital area. - With gynecological examination, vaginal walls and cervix can be explored. - US and vaginoscopy are important diagnostic tools for vaginal, uterine, and cervical IHs, while urine exam and cystoscopy are used when urethral and vesical IHs are suspected. - MRI and CT could be of use in selected cases. - For a conclusive pathologic diagnosis, an excisional biopsy is recommended when the diagnosis is uncertain.
New perspectives on therapy [7,11,13,15,17]	- Because of the rarity of the condition, there is no universally accepted method of treatment. - If the hemangioma is asymptomatic, it usually does not require treatment, but complications can call for additional care. Ulceration is the most common complication in the urogenital area and occurs in 5% of lesions. - Propranolol is the first-line treatment when needed, especially in highly symptomatic cases. Its mechanism is unclear, but might involve vasoconstriction, downregulation of growth factors including vascular endothelial growth factor (VEGF), and upregulation of cellular apoptosis. - Oral corticosteroids are used as second-line treatment instead of propranolol, because they have more side effects. For a small superficial lesion, intralesional corticosteroids could be an option. - Surgical excision is reported in the literature to be the best option for pedunculated lesions, with favorable symptomatic and aesthetic outcomes, and for lesions refractory to oral treatment, or that involve or compromise a vital organ or structure. - Alternative treatments are argon laser, cryosurgery, or pulse dye laser. However, the laser’s best results seem to be obtained in skin lesions less than 3 mm.

**Table 2:** Differential diagnosis of causes of vulvovaginal bleeding in pre-adolescent girls.

Differential Diagnosis of Bleeding in Pre-Adolescent Girls	
**Uterine IH**	**IH of the cervix, vagina, and urethra**
Ovulatory disfunction	Vaginal foreign body
Adenomyosis	Trauma
Neoplasm	Sexual abuse
Systemic bleeding disorders	Neoplasm
**Vulvar IH**	Endocrinopathy
Scratching lesions	McCune Albright syndrome
Juvenile lichen sclerosus	Systemic bleeding disorders
Dermatitis	Structural anomaly
Vulvovaginal infections	Vulvovaginitis caused by Shigella
Lipschutz ulcer	Stimulation of fetal endometrium by maternal estrogen in utero

## Data Availability

Not applicable.
